# Bronchitis

**Published:** 1877-04

**Authors:** Ross C. Russ

**Affiliations:** Hillsboro, O.


Bronchitis.—*By Ross C. Russ, M.D., Hillsboro, O.*—There is perhaps no disease that the human family is more likely to contract than that of bronchitis; no age or condition in life being exempt from it, and not unfrequently is very fatal both to the aged and to the infantile state.

It may also be of the most simple type of catarrhal inflammation to that of the most grave and protracted, not unfrequently compromising the life of the individual whom it attacks. The bronchio-pulmonary membrane, together with its glandular appendages, is the seat of the disease. Considering the normal histological anatomy of this important tissue, it is found to abound with numerous blood-vessels, lymphatics and mucous follicles, together with a flat expansion of epithelial structure, which not only lies as a covering of the mucous tissue, but also invests the follicles. The blood-vessels and lymphatics are exceedingly minute forming a closely set plexus. The incipient stage of the disease is active hyperemia of the minute capillary blood-vessels, thereby producing derangement of the healthy nutrition of the part, and at first producing a diminution of the follicular secretion, but sooner or later it becomes copious, in many cases, and especially in infants, by the augmentation of this secretion interterfering with the normal interchange of gases, and the child (unless relieved) soon becomes asphyxiated. Pathologically considered, while this disease is principally confined to the larger bronchial structure it is not so dangerous, but it too frequently occurs that the inflammation passes from above downward, in the same manner as an erysipelatous inflammation does on the cutaneous surface, producing thereby the more grave form of bronchial inflammation, and is usually designated by some medical writers as capillary bronchitis.

The secretion at this stage of the disease is moreover very copious and tenacious, and its elements, pathologically considered, consists of pus and fibrin, with epithelial cells, together with mucous from the larger tubes.

Bronchial inflammation may effect at this stage of the disease the parenchymatous tissue not as a direct inflammation of that part, but simply as a secondary result. The bronchi becoming obliterated by the excessive secretion, the air gradually finds its way out, though imperfectly, through the obstructed tubes. The expiratory effort, so long as there is air in the vesicles, constantly tends to dislodge the obstructing secretion; therefore, while the entrance of the air is constantly opposed to its exit, the air-vessels of the lobules which are beyond the place of obstruction become completely emptied and collapsed.

The result of the morbid process is solidification of the lobule or lobules of the parenchymatous tissue and not "lobular pneumonia" as hitherto taught by some pathological writers.

The condensed lobule or lobules do not remain in this state, for they suffer from innutrition and sooner or later become shriveled and atrophied, the small bronchial vessels becoming occluded undergo softening and liquefaction.

This stage of the disease not unfrequently puts on a chronic character, with harrassing cough, copious expectoration, and marked anemia simulating tuberculosis, but the differential disagnosis may be easily made out by the accute diagnostician from the physical signs, with the aid of the thermometer. According to my repeated examinations of cases in this disease and in tuberculosis I am convinced, that if there is daily exascerbation with an increase of body temperature, of at least not more than 1-5th of a degree, gradual loss of body and weight, with the physical sign of a peculiar dullness on percussion which is characteristic of tuberculosis; and the absence of the clear resonant sound which is characteristic of bronchial inflamation—discovered by a careful study of the diacrotic phenomena of the symptoms of bronchial inflamation and tuberculosis—the two diseases cannot easily be confounded.

Therefore in the enunciation of bronchial inflammation with its sequelæ, the definite law that a collapse of a lobule or lobules of one lung, not unfrequently eventuates in vesicular emphysema by an increase of volume of those portions of the pulmonary tissue to which the air has access, to compensate for the diminished volume of the collapsed, shriveled, atrophied, and very frequently liquefied portion of the lobule or lobules of the lungs should be considered.

At this stage of the disease the breathing is the greatest difficulty. The patient not unfrequently experiences a dread of suffocation, owing to the imperfect reception and distribution of atmospheric-air into the air-cells of the lungs.

The physical sign of the emphysematous complication of bronchitis is characterized by incompleteness of the expiratory act, the thorax remaining very prominent over the emphysematous lung. Upon percussion a resonant sound is elicited, and also the vesicular murmur is rendered indistinct, owing to the confinement of the air in the dilated air-vesicles.

The autopsical appearance of an emphysematous lung as a complication, is that the dilated air-vesicles may be seen clearly through the pulmonary pleura, and upon close examination of the parts, are found pale and quite white, the lobules dryer than normal and cannot be easily epmtied of air. The capillary vessels are obliterated, caused by the distension of the air-cells, thereby the lobule or lobules suffer by innutrition and ultimately soften and liquefy.

The treatment of bronchial inflammation should be conservative, it being self-limited and cannot be jugulated at once by any medical agent. It is, but too often, that the treatment of this disease is spoliative, depriving the patient of, and interfering with the healthy nutrition of the system. Therefore most, if not, all the medicinal agents designated as expectorants are contra-indicated—by interfering with the function of digestion and assimilation—thereby *lowering the vital forces* of the system, and ultimately producing innutrition. The tendency of bronchial inflammation is to debility; therefore it should be borne in mind, that the alimentary substances constituting the proper elements of nutrition, *i. e.* the aqueous, albuminous, saccharine, and oleaginous, should be given in proper combination and at stated intervals for the maintenance of nutrition. The saccharine and oleaginous should be in excess of the other alimentary substances, for they can be taken in smaller quantity and are greater fat producing materials. It is desirable that the reparation of the tissue should keep *pari passu* with their disintegration.

The medicinal treament for acute bronchial inflammation should be commenced with wine of ipecacuanha, given with the view to unload the bronchi of the excessive secretion, and to allay the irritability of the vagus nerve—but should only be given in the first stage. After the subsidence of the acute stage I have witnessed the best results from the following:

℞ Quinia sulphas, ʒiij.

Acid phos. dil., ℥ss.

Syr. Tolu, ℥ss.

M. Aquæ destil., ℥iij.

Sig.—A desert spoonful every four hours.

The dose should be increased or diminished according to age of the patient, for the very object which is to be obtained is to produce sedation over the turgid aud relaxed capillaries of the mucous tissue of the bronchi, at the same time increasing the tonicity of the part.

When this disease assumes a still more grave form with its sequelæ, solidification, and ultimately softening of the lobules of the lung tissue with great innervation of the system, the following should be given:

℞ Spts. vini gallici, ℥vj.

Glycerin, ℥ij.

M. Tinct. Hyoscyami, ʒiij.

Sig.—Teaspoonful every four or five hours.

The dose to be regulated according to age of the patient.

These medicinal agents soothe the harrassing cough, aid digestion, assist assimilation, and *prevent undue tissue change.*

There is perhaps no other disease in which counter-irritation has a better effect than in that of bronchial inflammation, when applied so as to produce a continuous fullness of the cutaneous capillaries of the chest. Baths or sponging with chloride of soda, with friction to the whole cutaneous surface judiciously regulated so as to keep up healthy action in the emunctories, and cause thereby elimination of the morbid products of the system, are highly indicated.

When the cough becomes so harrassing as to deprive the patient of rest—and it should be remembered that sleep has a great tendency to recuperate the enervated system—a hypodermic injection of sulph. morphia and sulph. atropia should be given at bed time. Laxatives only should be given to overcome constipation, and then the *Pulv. Glycerrhiza Comp.* of the *Prussian Pharmacopœia*—in doses of a teaspoonful in a wine glass of cold water until an alvine discharge is procured. If diarrhœa exist tannate of bismuth is indicated. These measures with the indications timely met, and the hygienic laws strictly observed and enforced throughout the whole progress of the disease will generally be sufficient for the management of bronchial inflammation and its concomitant sequelæ.—*Cincinnati Lancet and Observer.*

